# A Survey of Quality Assurance Practices in Biomedical Open Source Software Projects

**DOI:** 10.2196/jmir.9.2.e8

**Published:** 2007-05-07

**Authors:** Günes Koru, Khaled El Emam, Angelica Neisa, Medha Umarji

**Affiliations:** ^3^Children's Hospital of Eastern Ontario Research InstituteOttawaONCanada; ^2^University of Ottawa and Children's Hospital of Eastern Ontario (CHEO) Research InstituteOttawaONCanada; ^1^Department of Information SystemsUniversity of MarylandBaltimoreMDUSA

**Keywords:** Open source software, medical informatics, computational biology, information systems, software quality assurance, software/program verification, code inspections and walkthroughs, software reliability

## Abstract

**Background:**

Open source (OS) software is continuously gaining recognition and use in the biomedical domain, for example, in health informatics and bioinformatics.

**Objectives:**

Given the mission critical nature of applications in this domain and their potential impact on patient safety, it is important to understand to what degree and how effectively biomedical OS developers perform standard quality assurance (QA) activities such as peer reviews and testing. This would allow the users of biomedical OS software to better understand the quality risks, if any, and the developers to identify process improvement opportunities to produce higher quality software.

**Methods:**

A survey of developers working on biomedical OS projects was conducted to examine the QA activities that are performed. We took a descriptive approach to summarize the implementation of QA activities and then examined some of the factors that may be related to the implementation of such practices.

**Results:**

Our descriptive results show that 63% (95% CI, 54-72) of projects did not include peer reviews in their development process, while 82% (95% CI, 75-89) did include testing. Approximately 74% (95% CI, 67-81) of developers did not have a background in computing, 80% (95% CI, 74-87) were paid for their contributions to the project, and 52% (95% CI, 43-60) had PhDs. A multivariate logistic regression model to predict the implementation of peer reviews was not significant (likelihood ratio test = 16.86, 9 df, *P* = .051) and neither was a model to predict the implementation of testing (likelihood ratio test = 3.34, 9 df, *P* = .95).

**Conclusions:**

Less attention is paid to peer review than testing. However, the former is a complementary, and necessary, QA practice rather than an alternative. Therefore, one can argue that there are quality risks, at least at this point in time, in transitioning biomedical OS software into any critical settings that may have operational, financial, or safety implications. Developers of biomedical OS applications should invest more effort in implementing systemic peer review practices throughout the development and maintenance processes.

## Introduction

The importance of Information and Communications Technology (ICT) to the health care industry is rising as organizations attempt to find ways of reducing the costs of care and improving patient safety. However, in general, ICT adoption in health care is underfunded. For example, in Canada, the proportion of the 2003 national health care budget devoted to ICT was approximately 1.8% [1]. Canadian hospitals spend 1.8% to 2.5% of their budgets on ICT, which is low compared with other nations [2]. Recently ICT expenditures in Canadian hospitals have even been decreasing [3]. Other information-intensive sectors, such as banking and government, have ICT expenditures ranging from 9% to 13% of their operating budgets [1]. In the United States, the health care industry invests only 2% of gross revenue in ICT [4].

Therefore, cost is an important barrier to the adoption of information systems in health care [4,5]. This is motivating increased interest in open source (OS) software [6,7]. Surveys show that enterprises adopt OS primarily because they believe it is cheaper to acquire than the alternative solutions and believe it to have a lower total cost of ownership [21-24]. Many information technology (IT) managers also believe that OS is free [12]. It has been suggested that OS can address some of the biggest barriers to the adoption of electronic medical records (EMRs) by physicians [7,13]. Medicare in the United States has made a modified version of the OS Vista system freely available to all US medical practices [14]. In general, there is an increasing uptake of OS EMRs [15]. There also exist OS tools to support clinical trials [16] and imaging tools for radiologists [17]. In the bioinformatics domain, OS software has already gained popularity [21-24].

Software used in providing care is often safety and mission critical. There is evidence that software failures in clinical information systems and in medical devices have caused serious injuries and fatalities in patient populations [21-24]. Research and clinical applications are merging [25], highlighting the quality risks for research applications as well.

 Ensuring that software, including OS software, is of high quality and that the processes used to develop that software follow best software engineering practice is becoming a necessity. However, it has been suggested that the OS development model used for many bioinformatics products can have weaknesses in terms of quality assurance (QA) compared to the development of non-OS products that typically involve formal QA procedures [25].

In the commercial world, third-party process assessments (such as ISO 9000:2000 or Capability Maturity Model Integration [CMMI] [26]) are used to provide some assurance to end users that effective software engineering practices are in place during software development, in particular, QA practices. In an OS project, it is not possible to perform such assessments.

To facilitate the adoption of OS in the biomedical domain, and to understand the risks, if any, this paper reports on a Web survey of the QA practices used by the global biomedical OS developer community. The objectives of the survey were twofold:

1. To determine the rate at which two key QA practices, peer reviews and testing, are actually being used in biomedical OS projects
2. To determine whether the following factors have an impact on the implementation of these practices: experience of the developers, educational background of developers, size of the software product, and number of users

To our knowledge, this is the first study to focus on the QA practices of OS developers in the biomedical domain.

### Literature on QA Practices in OS Development

The current evidence on the QA practices implemented in general (nonbiomedical) OS projects was summarized. The two main QA activities we focused on were peer reviews and testing because they are the most commonly practiced in software development projects.

A literature search was conducted in the fall of 2005, primarily on EI Compendex and Inspec databases. The search was restricted to English articles published since 1995. To ensure that we cast a wide net, we used the term “open source software” for the search. The ACM digital library, IEEE Xplore, and SpringerLink were also searched. The reference lists from included studies were examined in an effort to identify additional articles and relevant dissertations.

A total of 2731 articles were initially identified. A level one screening based on the titles and abstracts removed articles that did not discuss software quality practices directly or indirectly. The second level of screening based on the full text included only articles that had original data on QA practices.

The quality of the studies varied greatly. There were no controlled experiments, and many of the original data came from surveys or qualitative studies. Below we summarize the evidence descriptively and then draw conclusions based on the consistency of evidence from multiple sources and using multiple methodologies.

### Peer-Review Practices

One of the fundamental assumptions for the success of OS is that there will be many developers looking over the source code. The popularized statement “given enough eyeballs, all bugs are shallow” captures that assumption [27]. The argument goes that because so many developers and users look at the source code of an OS application, this amounts to a large-scale peer-review process. Some authors are talking about millions of programmers performing peer review on OS code [28].

It is well established in software engineering that peer reviews are one of the best methods for defect detection [29]. In addition, in non-OS settings, the prospect of their code being scrutinized by their peers motivates programmers to be more careful [30,31].

Peer reviews are a relatively common practice in OS projects. For example, in the FreeBSD project, 57% of the respondents working on the project had distributed code for review in the month prior to being surveyed, and 86% of those who distribute code for review do get feedback from their peers [31]. In another survey, only 9% of OS developers indicated that all of their code was peer reviewed, but almost 50% indicated that most of their code was reviewed [32].

How does this compare to non-OS projects? One survey found that 52% of companies perform code reviews [33]. Another survey found that 71% of respondents in the United States use code reviews, around 82% in Europe, and 74% in Japan [34]. Therefore, there is evidence that a majority of non-OS software project developers also use some form of peer review.

In one study, the average number of reviewers per OS application was 1.3, including the author, and for larger projects, this number climbs to 7.5 reviewers [35]. The number of reviewers on larger OS projects is consistent with the numbers typically recommended in the software engineering literature for commercial (non-OS) projects. Porter et al [36] reported that peer reviews are usually carried out by a team of 4 to 6 inspectors. The recommended size for reviews defined by the Institute of Electrical and Electronics Engineers (IEEE) standard for software reviews and audits is 3 to 6 persons [37]. Ackerman et al [38] reported that reviews are conducted by at least 3 people, one of whom is the moderator who is responsible for the effectiveness of the examination. Lau et al [39] suggested 3-person reviews. Grady [40] stated an optimum size of 4 to 5 reviewers. Laitenberger and DeBaud [41] recommended 3 to 4 reviewers. They stated that there would be a ceiling effect after which an additional reviewer would not necessarily pay off in more defects being discovered. Fagan [42] recommended keeping review teams small, that is, 4 people. Weller [43] reported that 4-person teams were better at finding bugs than 3-person teams. Beyond 4 members, there is no performance improvement. Madachy et al [44] suggested an optimal size of 4 to 5 people for reviews. Gilb and Graham [45] mentioned a team size of 4 to 5 people to maximize the ability to find bugs. Strauss and Ebeneau [46] suggested a minimum review team size of 3 and a maximum of 7. In an experiment at the Jet Propulsion Laboratory, Kelly et al [47] stated that reviews are usually carried out by 6 people. Johnson [48] notes that there is widespread consensus that the review team size should never exceed 6 to 9 members. Industrial practice varies even within a single enterprise, for example, review teams between 4 and 12 are used at AT&T [49].

Two relevant characterizations of peer reviews are “ad-hoc” and “checklist” peer reviews. The former means that the reviewers do their best to find defects without any guidance. The latter means that a standardized checklist of common defects is used to guide the reviewers and to ensure that they look for all defect types. There is evidence that checklist-based techniques tend to find more defects than ad-hoc techniques [50]. There have been no studies on whether OS peer reviews utilize ad-hoc or checklist approaches.

### Testing Practices

Most OS projects do not perform extensive pre-release testing internally. The most common testing performed is unit testing. After that, a release candidate is made available, and the users try the release candidate and report its defects.

Below is a summary of the results of studies on pre-release testing activities and how well they are at finding bugs.

In general, pre-release testing is not a prevalent activity [51] and formal testing is not common [52]. The prospect of having their code peer reviewed makes OS developers more comfortable releasing their software with little testing [32], and more than 80% of OS developers in another survey responded that their products do not have testing plans [35]. This is consistent with other evidence showing that only 32% of projects had design documents [53] and that OS projects typically do not produce explicit requirements [54,55], making testing against requirements and design more difficult.

A majority of OS developers believe that anyone who downloads their code will check for and report bugs (implying that if there are no bug reports then no one has found anything) [35]. This indicates that testing effort by developers is minimal as they tend to rely on other people to look for defects.

The evidence on automated tool usage is mixed. A common testing tool is a debugger [35]. Only 48% of OS projects used a baseline test suite to support regression testing, and the percentage is only slightly higher as projects become large [53]. One early study reported that the Apache project had no regression or system test performed [54]. A more recent analysis did indicate that there was a regression test suite for Apache, but its use was not mandatory [56]. The Linux kernel did not go through pre-release testing by the developers, but rather users report defects in release candidates [56]. With the recent commercialization of Linux, considerable effort has been put into producing regression test suites [57], although an analysis of the test coverage of some of these test suites found that it was rather poor, with many critical subsystems having low or no coverage [58]. Subversion, another OS project, had an automated regression test suite [56]. Mozilla had dedicated test teams and test plans [54].

If we compare the above numbers with recent data on the implementation of regression testing in commercial software development, one can see that OS projects are somewhat lagging [34]. 

More than 50% of surveyed OS projects did not take advantage of code coverage metrics [35]. Only 5% of projects employed tools to measure test coverage [53], and almost 30% of projects had an (subjectively) estimated test coverage of less than 30% [53].

The above evidence indicates that developer pre-release testing in many OS projects is not a priority.

### Peer Reviews vs Testing

The argument has been made that the lack of developer pre-release testing is compensated for by the peer reviews that are conducted [52]. The problem in this argument is that there is substantial evidence that peer review will not find the same types of bugs (probabilistically) that testing will find [59-63]. Therefore, peer reviews and testing are not alternatives but rather are complementary. Consequently, doing only peer reviews will likely result in a smaller proportion of bugs being discovered than employing both peer reviews and testing.

One of the arguments made in support of OS quality is that OS programs tend to have a large number of end users who effectively beta test the software every time it is released by going into the code to identify where the bugs are and contributing the patches to correct these bugs. Such large-scale and free beta testing and debugging ensures that any defects that escape development will be discovered relatively quickly. The argument has some merit: it is known that defect discovery is affected by usage. Therefore, the more users a product has, the more defects will be discovered. However, as indicated below, few of the users actually contribute bug reports, and fewer still contribute bug fixes. Therefore, the number of users is not an appropriate reflection of the amount of debugging activity that is going on.

A study of the Apache project by the Software Engineering Institute [64] found that the majority (93%) of the changes (implementations, patches, and enhancements) were made by the core group of developers. The total number of people reporting bugs was 5116, but only 200 individuals actually contributed patches. The difficult and critical architectural changes were made by an even smaller subset of the core developer group [64].

Another investigation of Apache [65] found that more than 83% of the modification requests came from the top 15 developers, as did 88% of added lines of code and 91% of deleted lines of code. About 66% of bug fixes were produced by the top 15 developers, but 182 individuals submitted bug fixes out of 3060 who submitted bug reports [54]. The top 15 problem reporters submitted only 5% of the problem reports. These numbers indicate that new functionality is developed by a small core team, but there is wider participation in bug fixes. However, most of the people who report bugs do not actually submit any fixes. A small proportion of those who report problems are truly debuggers.

An interesting informal survey that was performed among Unix users (comprising researchers, staff, and students) at a computer science department asked if users had encountered bugs and if they reported them [66]. All of the respondents who had encountered bugs, some serious, did not bother to report them. To the extent that this behavior is common among technical users (Unix users tend to be rather technically savvy), many users will not bother reporting bugs even if they do discover them. In the case of Apache, it was estimated that less than 1% of all Apache users report problems [54].

In addition, most (80%) of the OS projects have less than 11 end users (where subscribers is used as a surrogate for users) [67]. Only 1% have more than 100 users [67]. A Pareto analysis of active projects on SourceForge found that half of the active projects had between 0 and 70 downloads per month [68] and that a very small number of projects are popular, with the vast majority not experiencing many downloads. Following a Pareto distribution means that the number of projects with more than a given number of downloads tails off exponentially.

Therefore, we can conclude that for most OS projects the number of users tends to be relatively small, the proportion of these users who report bugs is smaller, and the proportion of those who contribute patches are even smaller. It can be argued, then, that the extent of community debugging and beta testing is not that extensive in practice.

### Literature Review Summary

The existing evidence paints a decidedly mixed picture of the QA practices of OS projects. One can conclude that the better projects have practices that are, at best, comparable with non-OS projects. In general, peer reviews and testing, when practiced, tend to be minimal. Such practices are consistent with the results from studies directly measuring the postrelease quality of OS applications. An evaluation of the postrelease defect levels in Apache found that defect density was higher than a number of commercial products in the telecommunications field [54,65]. A recent study of FreeBSD also collected postrelease defect data [69], with mixed results when compared to non-OS products.

None of the studies that we found focused on biomedical applications—we do not know what the QA practices are for biomedical OS projects or what the resulting quality is. Therefore, it is unclear whether the conclusions from general OS studies can be extended to the biomedical domain.

## Methods

### Questionnaire Development

Our Web questionnaire was based on two previous general (nonbiomedical) OS developer surveys. The first survey was conducted by Stark [32,70] to understand the peer-review practices in OS projects. The second was performed by Zhao and Elbaum [35,53] and was geared toward understanding the QA activities in OS projects.

There were five sections in the questionnaire relevant to this paper: (1) respondent demographics, (2) project characteristics, (3) extent and nature of the implementation of peer reviews, (4) extent and nature of the implementation of testing, and (5) optional contact information (Multimedia Appendix 1).

A pilot study was conducted with software development staff at Georgetown Medical Center and the National Cancer Institute. A draft of the online questionnaire was sent to the two pilot sites, and comments were solicited on ease of understanding the questionnaire, the usability of the Web form, and the time it takes to complete. The questionnaire was revised based on this feedback.

### Survey Setup

The target population for the survey consisted of developers of biomedical OS projects. A project was considered an OS project only if its source code was publicly available. We made a list of biomedical OS projects by searching for OS projects in bioinformatics, medical informatics, and health care informatics domains. Biomedical OS projects were selected based on a Web search and expert inputs. The initial list was constructed based on the authors’ knowledge of OS applications. We then went to the OS project-hosting websites SourceForge [71] and FreshMeat [72] and identified additional projects. On SourceForge, we identified the projects listed under bioinformatics and medical science applications subcategories, which are under the Scientific/Engineering main project category. On FreshMeat, there was the exact same categorization, so we identified projects in the same way. We also used the BioMed Central website [73], where software developed for various biomedical research projects was available. Finally, we sent the project list to our colleagues and asked whether they would add any OS projects to our list. While creating the list of projects, we encountered some projects hosted on multiple sites with the same or similar names. Such duplicate project entries were eliminated. As a result of this in-depth search, we identified 229 projects (Multimedia Appendix 2).

After obtaining the list of projects, we started to identify the names and email addresses of the developers working on those projects using the following information sources:

1. Project websites: The names and email addresses of some developers were listed on project websites.
2. Source code repositories: OS projects often adopt a configuration management system, for example, Concurrent Versions Systems (CVS) or Subversion to allow developers to manage different versions of their source code files. Developers check in and check out source files to and from this repository. Usually, a log is kept in the source code repository for each checkin. The log includes the developer’s log-in name and email address. From these logs, we were able to identify some developers.
3. Defect databases: OS projects usually employ a defect-handling tool, Bugzilla, or a variant of it [74]. The defect records in these databases include rich information, such as the log-in names and email addresses of the developers assigned to solve the defects, which can be extracted.
4. Mailing lists: Developers in OS projects usually communicate using a designated mailing list. The emails sent to these lists are archived on the project websites. The header portions of the emails include the names and email addresses of developers.

Using multiple information sources allowed us to cross-validate the lists. Duplicate developer entries were eliminated. In our developer list, we did not include those who made minor contributions to the projects by occasionally fixing bugs, sending emails, or committing source code. As a result, our sample consisted of 750 developers heavily involved in the targeted projects. A small number of developers were involved in more than one project. We made it clear to those developers that they should base their questionnaire answers on a single project that we selected. Therefore, many developers from a project were allowed, but one developer could only answer for one project.

During and after the survey period, we took extensive precautions to maintain the confidentiality of the respondents’ records. Other than the one described above, no other prescreening or identification procedure was used.

The identified OS developers were sent an invitation email and a link to the final Web survey. After the first week, multiple reminders were sent to nonrespondents over a period of 6 weeks.

### Analysis Methods

The analysis was performed at two levels, the individual and project level. This suggests a multi-level approach to analysis. However, there were structural reasons why such a hierarchical modeling approach was not deemed appropriate in this case: most OS projects are small. In general, at least 5 respondents per project are recommended in order to model multiple levels and their interactions [75,76]. Of the 229 projects that were surveyed, only 23 (10%) had more than 5 developers. Of the 106 projects that we received responses for, 20 (19%) had more than one developer and only 2 (~2%) had more than 5 developers. Therefore, an alternative approach was necessary.

When reporting individual-level results, we will use all of the respondents’ records. For project-level analysis, individual responses in each project were aggregated. In the respondent database there were 138 observations and 106 projects. For aggregation, the most experienced developer’s (as determined by responses to the demographic questions) response was selected to represent the values for the project.

To address the first objective of the study (extent of use), descriptive statistics on the extent of use of the two QA practices were reported as proportions (percentages) with 95% confidence intervals.

To address the second objective (factors affecting the extent of use), multivariate logistic regression models [77] were developed for each of the main outcomes being investigated: implementation of peer reviews, measured by Q11 of the survey, and implementation of testing, measured by Q21 of the survey (see Multimedia Appendix 1). The unit of analysis was the project. The predictors consisted of the developer demographics and the project characteristics: years of programming experience (Q1 and Q2), whether the developer had a computing background (Q3), the number of users of the product (Q6), and the size of the product (Q9). It is reasonable to expect that the more experienced the developers, the more likely they will implement better software engineering practices. Also, we assumed that developers with a stronger computing background would be more likely to be associated with the implementation of key QA practices. The more users of the product, then the more individuals who are available to peer review, and this has been one of the core arguments made in support of OS software [27]. Finally, larger projects require the development team to impose more discipline and better practices to ensure the sustainability of the development effort (so that the project does not descend into a continuous cycle of bug fixes, each introducing even more bugs).

### Response Rate and Nonresponse Bias

There were no missing data since the Web survey tool made all questions mandatory. Therefore, all submitted forms were complete.

Since we performed our analysis separately at the individual and project level, we report the response rates for both. We received responses from 106 of the 229 projects contacted, which gave us a project-level response rate of 46.3%. Out of 750 developers contacted, 138 of them replied, which corresponded to a response rate of 18.4% at the individual level. Other Web surveys have shown a comparable response rate at the individual level [78]. The response rates in our survey were also comparable to previous surveys of OS developers, which ranged from 21% [79,80] to 34% [81].

Nonresponse bias was evaluated by comparing early respondents with late respondents [82]. We took the respondents who replied before the first reminder as early respondents. We compared the demographics of the early/late respondents and the characteristics of their projects using the Wilcoxon rank sum test [83]. None of the differences were significant at the .05 alpha level, indicating that the early and late respondents were identical in background characteristics.

### Summary

A summary of the survey setup and administration according to the CHERRIES guidelines [84] is provided in Multimedia Appendix 3.

## Results

### Background of Biomedical OS Developers

As can be seen from Table 1, 73% of the respondents (95% CI, 66,-81) had at least 5 years’ experience writing software. A large percentage of that experience was in the biomedical area. Therefore, one would expect these developers, in general, to have a good understanding of the computational needs in that domain.

In terms of project participation, 71% (95% CI, 63-79) of the respondents were participating in their projects part-time. Contrary to the assumption that OS developers do not receive compensation for their efforts, about half of all respondents were part-time and were paid by their employers for their contributions, and 80% (95% CI, 74-87) of the respondents received either part-time or full-time support for their development effort.

We also looked at the highest attained degree of the respondents, as summarized in Table 1. The biomedical OS developers were qualified professionals in their domain with 52% (95% CI, 43-60) of them having PhDs. A differentiation is made between those who had a computer science (or computer engineering) degree and those who did not (eg, biology, genetics, biochemistry, and physics). This distinction was based on the assumption that the computer science and computer engineering graduates would have a stronger grounding in software engineering practices than graduates of other disciplines. Almost three quarters of respondents (74%; 95% CI, 67-81) did not have a computing background.

**Table 1:** Developer education and experience (n = 138)

	**%**	**No.**
**Years of programming experience**		
< 1 year	2	3
1-5 years	25	34
> 5 years	73	101
**Years of experience in developing biomedical software**		
< 1 year	4	5
1-5 years	53	73
> 5 years	43	60
**Project participation level**		
Part-time, supported by employer	51	71
Part-time, personal time	20	27
Dedicated, full-time	29	40
**Highest academic degree and subject area**		
Bachelors in CS/CE	7	10
Masters in CS/CE	12	17
PhD in CS/CE	7	9
Bachelors in non-CS/CE	10	14
Masters in non-CS/CE	16	22
PhD in non-CS/CE	45	62
MD	3	4

CS/CE = computer science or computer engineering

Table 2 shows the experience level of the respondents in peer reviewing others’ code and in testing. Around 28% (95% CI, 21-36] of the developers had never peer reviewed others’ code, and approximately 19% (95% CI, 12-25) of them had received formal education in testing.

**Table 2:** Quality assurance experience of developers (n = 138)

	**%**		**No.**
**Years of experience in peer reviewing others’ code**			
None		28	39
< 1 year		12	17
1-5 years		28	38
> 5 years		32	44
**Formal education in testing**			
No		81	112
Yes		19	26

### Product and Project Characteristics

We found that 50% (95% CI, 40-60) of the products had at least 50 users. Therefore, it can be said that the quality of these biomedical products affects a large group of users. Approximately 63% (95% CI, 54-72) of the products are released at 6-month intervals or less, which is quite a rapid release cycle. Just under one third (30%; 95% CI, 21-39) of the products had been available for more than 3 years. Around a quarter (27%; 95% CI, 19-36) were larger than 50000 lines of code in size.

### Peer-Review Practices

Peer review is widely accepted to be one of the important strengths of the OS development model. However, for 63% (95% CI, 54-72) of the projects, peer review was not made an integral part of the development process, and peer review was never performed for 40% (95% CI, 30-49) of the projects.

Table 3 shows results for projects that did perform peer reviews (n = 64). We found that, in 81% (95% CI, 74-89) of those projects, peer reviews were never or only occasionally performed before the code is committed, and in 64% (95% CI, 55-73) of those projects, peer reviews were never or only occasionally performed before the product is released. A majority of projects (84%; 95% CI, 77-91) did not use checklists during their peer-review activities.

**Table 3:** Peer review practices at the project level, for projects that did perform some peer review (n = 64)

		**%**		**No.**
**Source code is ______ peer reviewed before commit.**				
Never	12		8	
Occasionally	69		44	
Half the time	3		2	
Frequently	8		5	
Almost always	8		5	
**Source code is ______ peer reviewed before product release.**				
Never	6		4	
Occasionally	58		37	
Half the time	6		4	
Frequently	10		6	
Almost always	20		13	
**Do you use a checklist for peer review?**				
No	84		54	
Yes	16		10	

Almost two fifths of all respondents (40%; 95% CI, 32-48) said that they never reviewed someone else’s code, 36% (95% CI, 28-44) never asked someone else to review their code, and 41% (95% CI, 32-49) said that no one reviewed their code. Of the survey respondents who asked others to review their code, 93% stated that three or fewer individuals do the review.

For those projects that did not perform peer-reviews (n = 42) we also asked for the reasons why. A high proportion (40%; 95% CI, 31-50) did not perform peer-reviews because there were other things to do (“work is too busy”), and 12% (95% CI, 6-18) were not because the developers believed that the code was already of sufficiently high quality that peer reviews were not needed. 17% were unsure how to review, and 7% said reviewing brings no beneft. For some projects, under the “Other” option, it was stated that the code was so large that it was not possible to apply peer reviews. In some projects, developers stated that they were the only developer, and they did not consider asking others to do peer reviews because they thought no one else would understand their code.

### Testing Practices

Testing was an integral part of the development process for 82% (95% CI, 75-89) of projects, and a regression test suite was run before every release for 58% (95% CI, 48-67) of the projects. The percentage of the projects for which a baseline test suite was used was 56% (95% CI, 46-65). Automated tools were used during development for only 25% (95% CI, 16-33] of the projects. One would conclude that automated testing is done after the development work is complete. Only 4% (95% CI, 0-7) of projects had automated test coverage tools. For the 21% (95% CI, 13-28) of the projects, the developer selected the “don’t know” option when asked about estimated code coverage. Only around 25% of the projects exceeded 80% code coverage.

As shown in Table 4, different types of testing are used in the projects. Unit testing is performed in 78% (95% CI, 70-86) of the projects. Approximately 70% of the projects (95% CI, 61-79) had unit testing performed half the time or more. Integration and system testing are also quite common. Testing is conducted continuously in 60% (95% CI, 51-70) of projects. In 32% (95% CI, 23-41) of projects, testing continues after releasing the software to specific users. Most defects that are fixed are found through testing rather than being discovered through usage.

**Table 4:** Testing practices and test results (n = 106)

		**%**	**No.**
**Do you perform ______?**			
Unit testing		78	83
Integration testing		64	68
System testing		75	80
System load and performance testing		45	48
Other		4	4
**How often do you unit test?**			
Never	8		9
Occasionally	22		23
Half the time	8		8
Frequently	17		18
Almost always	45		48
**What percentage of fixed defects is discovered by testing?**			
< 20%	16		17
20-40%	21		22
40-60%	23		24
60-80%	25		27
> 80%	15		16
**What percentage of fixed defects is discovered by users?**			
< 20%	47		50
20-40%	20		22
40-60%	15		16
60-80%	9		9
> 80%	9		9
**Testing is performed ______.**			
Continuously	60		64
Before release	57		60
After releasing to specific users	32		34
Randomly	29		31
Other	4		4

In Table 5, it can be seen that 80% (95% CI, 74-87) of the respondents spent less than 40% of their time on testing. The most common practices to generate test cases were the imitation of valid user behavior, generating failure inducing inputs, and using personal experience. Random testing and extreme load testing were not common.

**Table 5:** Testing practices at the individual level (n = 138)

	**%**		**No.**
**What percentage of the development time is spent on testing?**			
< 20%		44	61
20-40%		36	50
40-60%		14	19
60-80%		4	5
> 80%		2	3
**What strategies are adopted in choosing the test cases?**			
Provide inputs to imitate valid user behavior		82	113
Choose inputs most likely to cause failures		67	92
Choose inputs according to your experience		72	100
Use scripts to provide random inputs		20	28
Provide extreme values as inputs		46	63
Provide boundary conditions as inputs		43	59
Try extreme loads		28	38

### Multivariate Models

The objective of the multivariate models was to understand the factors that have an impact on the implementation of peer reviews and testing in biomedical OS projects. An initial analysis indicated that the two measures of years of programming experience (see Q1 and Q2 in Multimedia Appendix 1) were strongly correlated. We therefore constructed our models using Q1 only. The logistic regression model for the implementation of peer reviews was:

logit(Q10) ~ Q1 + Q3 + Q6 + Q9

And the logistic regression model for the implementation of testing was:

logit(Q21) ~ Q1 + Q3 + Q6 + Q9

The model variables are the answers to the following questions:

- Q1: The number of years of programming experience the respondent has.
- Q3: Whether the highest academic degree obtained by the respondent was in a computer science or a related area.
- Q6: The estimated current number of users of the OS product.
- Q9: The approximate size of the OS product.
- Q10: Whether peer review is an integral part of the project’s software development process.
- Q21: Whether testing was an integral part of the project’s software development process.

All independent variables except Q9 were ordinal. Therefore, repeated contrasts coding [85,86] was used to capture this ordering. For an independent variable with*k* ordered categories, *k* – 1 independent coding variables are used. The parameters for these coding variables represent the change in logit when the independent variable changes from one category to the next category.

The overall results for the main effect models are shown in Table 6, and the detailed parameter estimates in Table 7. An alpha level of .05 was used for all tests. These results indicate that none of main effect models are an improvement over the null model (with the intercept only).

An analysis of deviance comparing models with interaction effects versus main effects indicated that there were no interaction effects.

**Table 6:** The main effect models for the two outcome variables

**Model**	**Likelihood Ratio Test**	**Nagelkerke *R*^2^**
Peer review	16.84 (9 df); *P* = .051	0.201
Testing	3.34 (9 df); *P* = .95	0.051

**Table 7:** Detailed model parameter estimates for the two logistic regression models

**Variable**	Beta Coefficient^*^	Odds Ratio^*^	95% CI^*^	***P* value^†^**
**Peer Review Model**				
Intercept	8.76	–	–	.74
Q1: Programming experience (years)				
> 1-5 vs < 1	−0.15	0.86	−0.13 to 1.85	.80
5+ vs 1-5	−7.80	0.00	−0.02 to 0.02	.77
Q6: Number of users				
5-10 vs < 5	1.03	2.81	−0.5 to 6.11	.08
10-50 vs 5-10	0.014	1.01	−0.51 to 2.54	.98
50+ vs 10-50	−1.62	0.20	−0.17 to 0.57	.09
Q9: Size (lines of code)				
5000-20000 vs < 5000	−0.49	0.61	−0.15 to 1.38	.44
20000-50000 vs 5000-20000	1.21	3.37	−1.11 to 7.85	.07
> 50000 vs 20000-50000	−1.41	0.24	−0.07 to 0.56	.03
Q3: CS degree	0.33	1.39	0.05 to 2.73	.50
**Testing Model**				
Intercept	0.51	–	–	.79
Q1: Programming experience (years)				
1-5 vs < 1	−0.77	0.46	−0.3 to 1.23	.36
5+ vs 1-5	2.19	5.39	−8.35 to 19.13	.19
Q6: Number of users				
5-10 vs < 5	−0.17	0.84	−0.32 to 2.00	.81
10-50 vs 5-1-10	0.011	1.01	−0.67 to 2.70	.99
50+ vs 10-50	−0.33	0.72	−1.06 to 2.51	.79
Q9: Size (lines of code)				
5000-20000 vs < 5000	−0.21	0.81	−0.47 to 2.09	.79
20000-50000 vs 5000-20000	0.20	2.23	1.09 to 3.36	.80
> 50000 vs 20000-50000	−0.03	0.96	−0.44 to 2.37	.96
Q3: CS degree	−0.39	0.68	−0.08 to 1.44	.50

^*^Values are estimates.

^†^ *P* value is for the coefficient using the Wald Z statistic.

## Discussion

In summary, our descriptive results show that peer reviews have not been integrated into the development process for 63% (95% CI, 54-72) of the projects, while testing has been integrated into the development of 82% (95% CI, 75-89) of the projects. Approximately 74% (95% CI, 67-81) of developers did not have a background in computing, 80% (95% CI, 74-87) were paid for their contributions to the project, and 52% (95% CI, 43-60) had PhDs. A multivariate logistic regression model to predict the implementation of peer reviews was not significant (likelihood ratio test = 16.86, 9 df, *P* = .051) and neither was a model to predict the implementation of testing (likelihood ratio test = 3.34, 9 df, *P* = .95).

### Developer Background

The level of experience of the biomedical OS developers is consistent with the experience of developers of general (nonbiomedical) OS projects. Previous surveys found that developers have, on average, 11.86 years of programming experience [81]. However, compared to nonbiomedical OS projects, the biomedical developers tended to have less software engineering experience, but more advanced degrees. Previous surveys of general OS developers found that software engineers and programmers made up 43% of OS developers [87], 45.4% of OS developers were programmers [88], almost 80% of them worked in the IT sector [87], 58% were directly involved in the IT industry [81], 45% worked as professional programmers [81], and 51% had formal university level training in computer science and IT [81]. In addition, surveys of general OS developers found that a quarter of respondents had only high school or grammar school education [79,80], only 5% had PhDs, and 12% had masters degrees [87].

The lack of formal training and education in software development practices by the biomedical OS developers raises questions about the quality of the software being developed. However, our logistic regression models control for project characteristics and they did not find a relationship between programmer background/experience and the extent of implementation of peer reviews and testing. Therefore, it does not seem that the lack of formal training and education in software engineering has affected the implementation of good QA practices in these biomedical OS projects.

The percentage of developers financially supported to develop the biomedical OS applications is much larger than that seen in nonbiomedical OS projects. For example, other surveys of general OS developers found that 16% [79,80], 20% [87,89], 30% [88], and 40% [81] of developers are paid for their OS contributions. In our survey, we found around 80% of developers were being paid for their contributions. It is not clear from our results whether there were any commercial interests financing such development work.

### Implementation of Peer Reviews

Even though extensive peer reviews are often claimed to be one of the main advantages of the OS development paradigm, our results indicate that this practice is not prevalent, with the majority of projects not undergoing peer review on a consistent basis, and two fifths never doing so. This finding is somewhat consistent with peer review of general (nonbiomedical) OS projects, which was summarized earlier in the paper.

For projects that perform peer reviews, how well is the peer review implemented? A key performance measure for a peer review is the proportion of defects that it finds in the code (this is known as the effectiveness of the peer review). There are a number of factors that will have an influence on that: the number of reviewers, the capability of the reviewers (often measured by the proportion of defects that an individual reviewer can find), and the reading technique that is used.

We found in our survey that a maximum of three reviewers review a piece of code when it is peer reviewed. If we assume that reviewers are independent, which is a reasonable assumption in a distributed development project with little to no face-to-face interaction, a basic model of the probability of finding a defect is given by 1 – (1 – *p*)^i^ where *p* is the probability that an individual reviewer will find a defect (assuming that all reviewers are equally capable) and *i* is the number of reviewers. A previous review of the literature [90] determined that the average probability of finding a defect through code peer review was 0.57 and the maximum (or best-in-class) was 0.7. These numbers came from industrial, non-OS projects.

**Figure 1:**
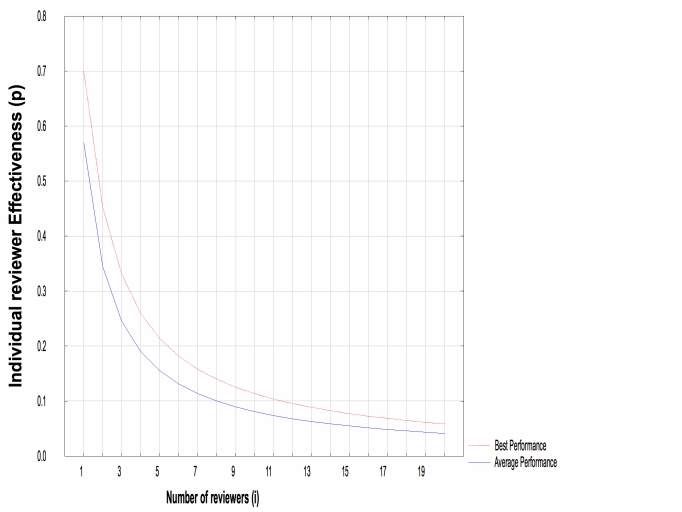
Relationship between the number of reviewers and individual effectiveness when the team effectiveness is fixed at 0.57 (average performance) and 0.7 (best performance)

Figure 1 shows the theoretical relationship based on the above model between the number of reviewers (x-axis) and individual reviewer effectiveness (y-axis) if we fix the overall peer review effectiveness at 0.57 and at 0.7. We can see that if a team of at most three programmers reviews a code snippet, then they would each have to have a *p* of at least 0.24 to achieve average performance, and a *p* of 0.33 to achieve maximum (best-in-class) performance. These would be the minimal reviewer capabilities for a team of three to achieve the average and maximum peer review effectiveness reported for non-OS projects, respectively. Are these minimal capabilities plausible, that is, is it plausible that the biomedical OS reviewers achieve defect detection effectiveness levels that high?

The literature on individuals’ effectiveness in code reviews can be examined to answer this question. Wohlin et al [91] created virtual review teams using the data sets collected from the literature, and they found that the effectiveness of individual reviewers had a median value close to 0.25. In another study, Runeson and Wohlin [92] reported that the defect detection rates observed during their experiments involving students and professionals had a median value of 0.31. Land et al [93] reported that, in their experiment, an individual detected an average of 5.51 out of 33 defects (effectiveness of approximately 0.17). Porter et al [94] reported the true positive ratios as a measure of effectiveness instead of the detection rates. On average, 13% of the reported issues turned out to be true defects. Dunsmore et al [50] conducted a code inspection experiment for Java programs in which they reported both detection ratio and false positives. The detection ratio for checklist-based reading was 52.14%, and the false positive rate was 24.50%. In this experiment, the subjects reviewed 200 lines of Java code with which they had familiarity from previous exercises, and they were provided with class diagrams and natural language specifications of all systems. In Land et al’s experiment, the subjects were provided with flowcharts, pseudo-code, and other code overview documents. In realistic OS development environments, such aids are less likely to be available. Therefore, it will be more difficult for the OS developers to reach the effectiveness levels mentioned in these studies.

Even if it is assumed that it is plausible for a biomedical OS peer reviewer to achieve average effectiveness rates as high as those achieved by non-OS peer reviewers, it would be less likely that OS projects would achieve the maximum or best-in-class effectiveness rates seen in non-OS projects. In addition, if the number of reviewers dips below three, then it is not likely that the effectiveness of the peer review would match the average performance of the non-OS projects.

That very few of the projects use checklists during the code reading also indicates that the effectiveness of these peer reviews will be relatively low.

### Implementation of Testing

How do testing activities in biomedical OS projects compare to testing in general OS projects? Zhao and Elbaum [53] noted that 58% of the generic OS projects spent more than 20% of their development time in testing. We found that 51% of the biomedical OS projects spent more than 20% of their time in testing. In generic OS projects, almost 30% had below 30% code coverage [53]. For 35.85% of the biomedical OS projects, the coverage was either not known or it was estimated as below 20%. The use of a regression test suite in biomedical OS projects was almost 58% compared to 3% in very large generic OS projects. In summary, testing activities in biomedical OS projects showed similarity to those in the generic OS projects. However, compared to the closed-source projects [34], there is still room for improvement.

### Summary

The major QA risk item in biomedical OS software projects is that code peer reviews are not systematically performed. Peer reviews are an important mechanism to find bugs in software, and they are complementary to testing.

Applications that collect and manage patient data can lead to negative financial and patient safety outcomes if they have subtle defects in them. OS developers therefore need to focus more attention on integrating peer review into their development practices to ensure that good software engineering practices are systematically employed. Acquirers of biomedical OS applications need to ensure that some form of peer review is being consistently practiced in the software projects producing and maintaining the applications that they deploy.

### Limitations

We highlight two limitations in this study. The first is the low response rate, although the response rate that we obtained is consistent with other studies in the same domain. In addition, we found no evidence of nonresponse bias. Second, there are many OS projects that were not included in our sample, and it is plausible that the ones we focused on tended to be the smaller ones.

### Conclusions

The OS software development paradigm has been suggested as a new and more effective way to develop high-quality software. In many health-related settings, ranging from care to research, it is important to ensure that software failures are minimized.

In this paper we performed a survey of biomedical OS software developers to understand their QA practices. Our results indicate that the major risk item in biomedical OS projects, from a software quality perspective, is the (lack of) implementation of peer reviews. Furthermore, when they are implemented, their performance is below what would be considered best practice. We also found that most of the developers did not have computer science or computer engineering training or education that could provide them with software engineering background. On the other hand, we found no evidence linking the lack of computer science or engineering background with the extent of implementation of peer reviews and testing, indicating that such background variables do not have an impact.

These results highlight some risk from transitioning biomedical OS applications into environments where they may have an impact on patient safety. For this transition to occur, it is important that better peer review practices be put in place. To the extent possible, developers of biomedical OS software should rely on Food and Drug Administration regulations and guidelines, such as 21 CFR Part 11, as well as professional society publications [95] documenting what are considered to be best software engineering practices.

## Multimedia Appendix 1

Survey form
						

## Multimedia Appendix 2

List of OS projects contacted for survey
						

## Multimedia Appendix 3

CHERRIES [84] summary for the Web survey
						

## Abbreviations

EMR: electronic medical record
ICT: information and communications technology
IT: information technology
OS: open source
QA: quality assurance

## References

[1] Prada G, Roberts G, Vail S, Anderson M, Down E, Fooks G, Howaston A, Grimes K, Morgan S, Parent K, Sinclair D, Thompson V, Yalnizyan A (2004). Understanding Health Care Cost Drivers and Escalators.

[2] Prada G, Grimes K, McCleery A, Nguyen D, Pomey MP, Reed V, Stonebridge C, Roberts G (2004). Challenging Health Care System Sustainability: Understanding Health System Performance of Leading Countries.

[3] Irving R (2003). Report on IT in Canadian Hospitals. Canadian Healthcare Technology.

[4] Raymond B, Dold C (2002). Clinical Information Systems: Achieving the Vision.

[5] Bates D, Ebell M, Gotlieb E, Zapp J, Mullins H (2003). A proposal for electronic medical records in US primary care. J Am Med Inform Assoc.

[6] Goulde M, Brown E (2006). Open Source Software: A Primer for Health Care Leaders.

[7] Kantor Gareth S, Wilson Wayne D, Midgley Adrian (2003). Open-source software and the primary care EMR. J Am Med Inform Assoc.

[8] Giera J (2004). The Costs and Risks of Open Source.

[9] Hunt F, Probert D, Barratt S (2003). Adopting new technology: the case of open source software at Marconi. The 12th International Conference on Management of Technology (IAMOT May 12-15 2003) Nancy, France.

[10] Dal Molin J (2003). Open Source Software in Canada. A Collaborative Fact Finding Study. e-cology Corporation.

[11] Dedrick J, West J (2004). An exploratory study into open source platform adoption. Proceedings of the 37th Hawaii International Conference on System Sciences.

[12] DiDio L (2004). Linux, Unix and Windows TCO Comparison, Part 1.

[13] Valdes Ignacio, Kibbe David C, Tolleson Greg, Kunik Mark E, Petersen Laura A (2004). Barriers to proliferation of electronic medical records. Inform Prim Care.

[14] Kolata G (2005). In unexpected Medicare benefit, US will offer doctors free electronic records system. New York Times Jul 21.

[15] Goldstein D, Ponkshe S, Maduro R (2004). Analysis of Open Source Software (OSS) and EHRs: Profile of Increasing Use of OSS in the Federal Government and Healthcare.

[16] Elsner C, Egbring M, Kottkamp H, Berger T, Zoller S, Hinricks G (2003). Open source or commercial products for electronic data capture in clinical trials? A scorecard comparison. Comput Cardiol.

[17] Erickson Bradley J, Langer Steve, Nagy Paul (2005). The role of open-source software in innovation and standardization in radiology. J Am Coll Radiol.

[18] Gentleman RC, Carey VJ, Bates DM, Bolstad B, Dettling M, Dudoit S, Ellis B, Gautier L, Ge Y, Gentry J, Hornik K, Hothorn T, Huber W, Iacus S, Irizarry R, Leisch F, Li C, Maechler M, Rossini AJ, Sawitzki G, Smith C, Smyth G, Tierney L, Zha J, Yang JYH (2004). Bioconductor: open software development for computational biology and bioinformatics. Genome Biol.

[19] Stajich J (2002). The Bioperl Project: A Look Ahead. Talk presented at: Bioinformatics Open Source Conference; August 1-2, Edmonton, Canada.

[20] Pocock M (2002). BioJava Toolkit Progress. Talk presented at: Bioinformatics Open Source Conference; August 1-2,.

[21] McCormick J, Gage D Cincinnati Children's Hospital: Shots in the Dark. Baseline. 2004 Aug 1. http://www.baselinemag.com/article2/0,1540,1655082,00.asp.

[22] Gage D, McCormick J Why Software Quality Matters: 'We Did Nothing Wrong'. Baseline. 2004 Mar 4. http://www.baselinemag.com/article2/0,1397,1544403,00.asp.

[23] Basili V, Belady L, Boehm B, Brooks F, Browne J, DeMillo R, Feldman S, Green C, Lampson B, Lawrie D, Leveson N, Lynch N, Weiser M, Wing J (1999). Final report: NSF workshop on a software research program for the 21st century. Software Eng Notes.

[24] Report of the Inquiry into the London Ambulance Service (1993). http://www.cs.ucl.ac.uk/staff/A.Finkelstein/las/lascase0.9.pdf.

[25] van Heusden P (2004). Applying Software Validation Techniques to Bioperl. Talk presented at: Bioinformatics Open Source Conference; July 29-30,.

[26] Konrad M, Shrum S, Chrissis M-B (2003). CMMI: Guidelines for Process Integration and Product Improvement.

[27] Raymond ES (1999). The Cathedral and the Bazaar: Musings on Linux and Open Source by an Accidental Revolutionary.

[28] Greiner S, Boskovic B, Brest J, Zumer V (2003). Security issues in information systems based on open source technologies. EUROCON.

[29] El Emam K (2001). Software Inspection Best Practices. In: Agile Project Management Advisory Service, Executive Report. Volume 2. Number 9.

[30] Bagchi S, Madeira H Open source software - a recipe for vulnerable software, or the only way to keep the bugs and the bad guys out? Panel position statement presented at 14th International Symposium on Software Reliability Engineering (ISSRE) 2003.

[31] Jorgensen N (2001). Putting it all in the trunk: incremental software development in the FreeBSD open source project. Inform Syst J.

[32] Stark J (2002). Peer reviews as a quality management technique in open-source software development projects. European Conference on Software Quality.

[33] Maccormack A, Kemerer C, Cusumano M, Crandall B (2003). Trade-offs between productivity and quality in selecting software development practices. IEEE Software.

[34] Cusumano M, MacCormack A, Kemerer C, Randall B Software development worldwide: the state of the practice. IEEE Software 2003.

[35] Zhao L, Elbaum S (2000). A survey on quality related activities in open source. Software Eng Notes.

[36] Porter A, Siy H, Toman C, Votta L (1997). An experiment to assess the cost-benefits of code inspections in large scale software development. IEEE T Software Eng.

[37] STD I (1989). IEEE Standard for Software Reviews and Audits. ANSI/IEEE STD 1028-1988.

[38] Ackerman AF, Buchwald LS, Lewski FH (1989). Software inspections: an effective verification process. IEEE Software.

[39] Lau L, Jeffery R, Sauer C (1996). Some Empirical Support for Software Development Technical Reviews.

[40] Grady RB (1992). Practical Software Metrics for Project Management and Process Improvement.

[41] Laitenberger O, Debaud JM (2000). An encompassing life-cycle centric survey of software inspection. J Syst Software.

[42] Fagan ME (1976). Design and code inspections to reduce errors in program development. IBM Syst J.

[43] Weller EF (1993). Lessons from three years of inspection data. IEEE Software.

[44] Madachy R, Little L, Fan S (1993). Analysis of a successful inspection program. Proceedings of the 18th Annual NASA Software Engineering Laboratory Workshop.

[45] Gilb T, Graham D (1993). Software Inspection.

[46] Strauss S, Ebenau R (1994). Software Inspection Process.

[47] Kelly J, Sheriff J, Hops J (1992). An analysis of defect densities found during software inspections. J Syst Software.

[48] Johnson P (1998). Reengineering inspection. Comm ACM.

[49] Eick S, Loader C, Long M, Votta L, Weil SV (1992). Estimating software fault content before coding. Proceedings of the 14th International Conference on Software Engineering.

[50] Dunsmore A, Roper M, Wood M (2003). The development and evaluation of three diverse techniques for object-oriented code inspection. IEEE T Software Eng.

[51] Halloran T, Scherlis WL (2002). High quality and open source software practices. Position paper presented at: The Second Workshop on Open Source Software Engineering, International Conference on Software Engineering; May 19-25,.

[52] Gacek C, Arief B (2004). The many meanings of open source. IEEE Software.

[53] Zhao L, Elbaum S (2003). Quality assurance under the open source development model. J Syst Software.

[54] Mockus A, Fielding R, Herbsleb J (2002). Two case studies of open source software development: Apache and Mozilla. ACM T Softw Eng Meth.

[55] Sharma S, Sugumaran V, Rajagoplan B (2002). A framework for creating hybrid open source software communities. Inform Syst J.

[56] Erenkrantz J (2003). Release management within open source projects. Proceedings of the Third Workshop on Open Source Software Engineering, Portland, OR.

[57] Thomas C (2003). Improving verification, validation, and test of the Linux kernel: the Linux stabilization project. Proceedings of the Third Workshop on Open Source Software Engineering.

[58] Iyer M (2002). Analysis of Linux test project's kernel code coverage.

[59] Myers G (1978). A controlled experiment in program testing and code walkthroughs/inspections. Comm ACM.

[60] Basili V, Selby R (1987). Comparing the effectiveness of software testing strategies. IEEE T Software Eng.

[61] Kamsties E, Lott C (1995). An empirical evaluation of three defect-detection techniques. Proceedings of the 5th European Software Engineering Conference, Sitges, Spain, September 25-28,.

[62] Wood M, Roper M, Brooks A, Miller J (1997). Comparing and combining software defect-detection techniques. Proceedings of the 6th European Conference on Software Engineering.

[63] Jalote P, Haragopal M (1998). Overcoming the NAH Syndrome for Inspection Deployment. Proceedings of the International Conference on Software Engineering.

[64] Hissam S, Weinstock C, Plakosh D, Asundi J (2001). Perspectives on Open Source Software. CMU/SEI-2001-TR-019.

[65] Mockus A, Fielding RT, Herbsleb J (2000). The Apache server.. Proceedings of the 22nd International Conference on Software Engineering.

[66] Miller B, Fredriksen L, So B (1990). An Empirical Study of the Reliability of Unix Utilities. Comm ACM.

[67] Capiluppi A, Lago P, Morisio M (2003). Characteristics of open source projects. Proceedings of the Seventh European Conference on Software Maintenance and Engineering;.

[68] Hunt F, Johnson P (2003). On the Pareto Distribution of Sourceforge Projects.

[69] Dinh-Trong T, Bieman J (2005). The FreeBSD project: a replication case study of open source development. IEEE T Software Eng.

[70] Stark JE (2001). Peer Reviews in Open-Source Software Development.

[71] SourceForge Home page. sourceforge.net.

[72] FreshMeat Home page. freshmeat.net.

[73] BioMed Central Home page. biomedcentral.com.

[74] Koru AG, Tian J (2004). Defect Handling in Medium and Large Open Source Projects. IEEE Software.

[75] Kreft I, Yoon B (1994). Are multilevel techniques necessary? An attempt at demystification. Annual Meeting of the American Educational Research Association; April 4-8,.

[76] Maas C, Hox J (2005). Sufficient Sample Sizes for Multilevel Modeling. Methodology.

[77] Hosmer D, Lemeshow S (1989). Applied Logistic Regression.

[78] Schonlau M, Fricker RD, Elliott MN (2002). Conducting Research Surveys via E-mail and the Web.

[79] Hars A, Ou S (2002). Working for free? Motivations for participating in open source projects. Int J Electron Comm.

[80] Hars A, Ou S (2001). Working for free? Motivations of participating in open source projects. Proceedings of the 34th Hawaii International Conference on System Sciences.

[81] Lakhani K, Wolf R (2005). Why hackers do what they do: understanding motivation and effort in free/open source software projects.

[82] Lindner JR, Murphy TH, Briers GE (2001). Handling nonresponse in social science research. J Agr Educ.

[83] Siegel S, Castellan J (1988). Nonparametric Statistics for the Behavioral Sciences.

[84] Eysenbach Gunther (2004). Improving the quality of Web surveys: the Checklist for Reporting Results of Internet E-Surveys (CHERRIES). J Med Internet Res.

[85] Serlin R, Levin J (1985). Teaching how to derive directly interpretable coding schemes for multiple regression analysis. J Educ Stat.

[86] Wendorf C (2004). Primer on multiple regression coding: Common forms and the additional case of repeated contrasts. Understand Stat.

[87] Robles G, Scheider H, Tretkowski I, Weber N (2001). Who is doing it? A research on Libre Software developers.

[88] Lakhani K, Wolf B, Bates J, DiBona C (2002). The Boston Consulting Group hacker survey. O'Reilly Open Source Conference; July 22-26,.

[89] Hertel G, Niedner S, Herrmann S (2003). Motivation of software developers in open source projects: an Internet-based survey of contributors to the Linux kernel. Res Pol.

[90] Briand L, El Emam K, Laitenberger O, Fussbroich T (1998). Using simulation to build inspection efficiency benchmarks for development projects. Proceedings of the 20th International Conference on Software Engineering.

[91] Wohlin C, Aurum A, Petersson H, Shull F, Ciolkowski M (2002). Software inspection benchmarking - a qualitative and quantitative comparative opportunity. METRICS '02 Proceedings of the 8th International Symposium on Software Metrics.

[92] Runeson P, Wohlin C (1998). An experimental evaluation of an experience-based capture-recapture method in software code inspections. Empir Softw Eng.

[93] Sauer C, Jeffery R, Land LPW (1997). Validating the defect detection performance advantage of group designs for software reviews: report of a laboratory experiment using program code. ESEC '97/FSE-5: Proceedings of the 6th European conference held jointly with the 5th ACM SIGSOFT international symposium on Foundations of software engineering.

[94] Porter A, Siy H, Toman C, Votta L (1995). An experiment to assess the cost-benefits of code inspections in large scale software development. Proceedings of the 3rd ACM SIGSOFT Symposium on Foundations of Software Engineering.

[95] The Good Automated Manufacturing Practice (GAMP) Guide for Validation of Automated Systems in Pharmaceutical Manufacture (2002).

