# Supplementary material for: A Survey of Quality Assurance Practices in Biomedical Open Source Software Projects
Source: J Med Internet Res. 2007 May 7;9(2):e8. doi: 10.2196/jmir.9.2.e8 (PMC1874720; doi:10.2196/jmir.9.2.e8)
Supplement: Supplementary file 1 [file jmir_v9i2e8_app1.pdf]

## Multimedia Appendix 1. Survey Form[*]

<u>Demographics and Background</u>

1. How many years of programming experience do you have?
   - ☐ <1 year
   - ☐ 1-5 years
   - ☐ 5+ years

2. How many years have you been involved in developing bioinformatics/biomedical software?
   - ☐ <1 year
   - ☐ 1-5 years
   - ☐ 5+ years

3. Highest academic degree and area: (e.g., M.S in Computer Science)

   ________________________

<u>Product Characteristics</u>

4. Please specify the name of this open source product:

   ________________________

5. What level of participation do you have in developing this product ?
   - ☐ Dedicated full-time
   - ☐ Part-time, supported by employer
   - ☐ Part-time, personal time

6. What is the estimated current number of users of this product?
   - ☐ 1-5
   - ☐ 5-10
   - ☐ 10-50
   - ☐ 50+

7. How often are the product releases (on an average)?
   - ☐ Every week
   - ☐ Every month
   - ☐ Every quarter
   - ☐ Every six months
   - ☐ Every year
   - ☐ Only one release at this time

8. How long has this product been available to users (i.e., since the first stable release)?

---

[*] Some survey questions were not analyzed in this paper, and hence the relevant questionnaire sections have been excluded.

- ☐ < 6 months
- ☐ 6 months - 1 year
- ☐ 1 - 3 years
- ☐ > 3 years

9. What is the approximate size of this product?
   - ☐ < 5,000 LOC
   - ☐ 5,000 – 20,000 LOC
   - ☐ 20,000 - 50,000 LOC
   - ☐ >50,000 LOC

Peer Reviews

10. Is peer-review an integral part of your software development process?
    - ☐ Yes
    - ☐ No

11. When are peer review performed?
    - ☐ Never
    - ☐ Before any source code is committed to the code base (continuously)
    - ☐ Before product release
    - ☐ Randomly
    - ☐ Other: _______________

12. In general, how often is code peer reviewed before it is committed to the code base?
    - ☐ Almost always
    - ☐ Frequently
    - ☐ Half the time
    - ☐ Occasionally
    - ☐ Never

13. In general, how often is code peer reviewed before product release?
    - ☐ Almost always
    - ☐ Frequently
    - ☐ Half the time
    - ☐ Occasionally
    - ☐ Never

14. How frequently do you peer review other people's code in this project?
    - ☐ Almost always
    - ☐ Frequently
    - ☐ Half the time
    - ☐ Occasionally

- ☐ Never

15. How often do you ask that others peer review your code?
    - ☐ Almost always
    - ☐ Frequently
    - ☐ Half the time
    - ☐ Occasionally
    - ☐ Never

16. If code is not peer reviewed, what is the reason?
    - ☐ It is considered unnecessary as the code is usually of high quality
    - ☐ Work is too busy to review code
    - ☐ Reviewing code brings no benefit
    - ☐ Unsure how to review to best effect
    - ☐ Other: _______________

17. How much experience do you have of peer-reviewing other people's code ?
    - ☐ <1 year
    - ☐ 1-5 years
    - ☐ 5+ years
    - ☐ None

18. Do you use a checklist for peer review?
    - ☐ Yes
    - ☐ No

19. On average, how many people typically peer review your code snippets?
    - ☐ 1
    - ☐ 2 - 3
    - ☐ 5-10
    - ☐ 10+
    - ☐ None

<u>Testing</u>

20. Have you received any formal education/training in software testing?
    - ☐ Yes (Please specify)
    - ☐ No

21. Is testing an integral part of your open source development process?
    - ☐ Yes
    - ☐ No

22. Testing is performed:
    - ☐ Continuously (e.g. before any source code is committed to the code base)
    - ☐ Before product release (alpha testing)

- ☐ After product is released to specific users
- ☐ Randomly
- ☐ Other: _______________

23. Do you perform?
    - ☐ Unit testing (testing whether individual modules function as expected)
    - ☐ Integration testing (testing a whether group of modules function as expected)
    - ☐ System testing (testing high-level usage scenarios)
    - ☐ System load and performance testing
    - ☐ Other: _______________

24. How often do you unit test the software that you write?
    - ☐ Almost always
    - ☐ Frequently
    - ☐ Half the time
    - ☐ Occasionally
    - ☐ Never

25. What strategy do you adopt in choosing your test cases?
    - ☐ Provide inputs trying to imitate valid user behavior
    - ☐ Choose those inputs most likely to cause failure
    - ☐ Choose inputs according to your experience
    - ☐ Use script to provide random values as inputs
    - ☐ Provide extreme values as inputs
    - ☐ Provide boundary conditions as inputs
    - ☐ Try extreme loads on the system (e.g., many users and large volumes of data)
    - ☐ Other

26. What percentage of your time is spent on testing
    - ☐ <20%
    - ☐ 20% - 40%
    - ☐ 40% - 60%
    - ☐ 60% - 80%
    - ☐ >80%

27. Do you contribute your test cases to a regression test suite?
    - ☐ Yes
    - ☐ No

28. Do you have a “baseline” test suite that you re-run in your software before every release?
    - ☐ Yes
    - ☐ No

29. What percentage of the source code is covered by the testing activity?
   - ☐ <20%
   - ☐ 20% - 40%
   - ☐ 40% - 60%
   - ☐ 60% - 80%
   - ☐ >80%
   - ☐ Don't know

30. The coverage information in the previous question was based on:
   - ☐ Personal estimation
   - ☐ Reports by coverage tool (please specify name: ____________________)

31. What percentage of the defects that you fix are discovered during your testing?
   - ☐ <20%
   - ☐ 20% - 40%
   - ☐ 40% - 60%
   - ☐ 60% - 80%
   - ☐ >80%

32. What percentage of the defects that you fix are discovered by users?
   - ☐ <20%
   - ☐ 20% - 40%
   - ☐ 40% - 60%
   - ☐ 60% - 80%
   - ☐ >80%

33. Do you use testing tools during your development?
   - ☐ Yes (Please give name: ___________________)
   - ☐ No

<u>Suggestions, Comments, and Feedback</u>

Please do let us know about your suggestions and comments about this survey.

_______________________________________________________________________

_______________________________________________________________________

Would you consent to be contacted later via e-mail if we need some additional information about your response?
   - ☐ Yes
   - ☐ No
   - ☐ No answer
