# Supplementary material for: A Survey of Quality Assurance Practices in Biomedical Open Source Software Projects
Source: J Med Internet Res. 2007 May 7;9(2):e8. doi: 10.2196/jmir.9.2.e8 (PMC1874720; doi:10.2196/jmir.9.2.e8)
Supplement: Supplementary file 2 [file jmir_v9i2e8_app2.pdf]

Multimedia Appendix 2. List of OS Projects Contacted for Survey

**Project Name**	**Web Page**
3D Slicer	http://www.slicer.org/
Apollo	http://www.fruitfly.org/annot/apollo/
Biobuilder	http://www.biomedcentral.com/1471-2105/5/43
Bioconductor	http://www.bioconductor.org
Biojava	http://www.biojava.org/
Biomail Scientific References Automation	http://biomail.sourceforge.net/biomail/index.html
Bioperl	http://bioperl.org/
Biophp	http://biophp.org
Biopython	http://www.biopython.org/
Bioquery	http://www.bioquery.org/
Biowarehouse	http://bioinformatics.ai.sri.com/biowarehouse/
Cd-Hit Sequence Clustering Software	http://bioinformatics.org/cd-hit/
Chemistry Development Kit	http://almost.cubic.uni-koeln.de/cdk/
Coasim	http://www.daimi.au.dk/~mailund/CoaSim/
Cytoscape	http://www.cytoscape.org
Das	http://biodas.org/
E-Cell System	http://sourceforge.net/projects/ecell/
Emboss	http://emboss.sourceforge.net/
Ensemble	http://www.ensembl.org/info/software/versions.html
Eviewbox Dicom Java Project	http://sourceforge.net/projects/eviewbox/
Freemed Project	http://bioinformatics.org/project/?group_id=298
Ghemical	http://www.bioinformatics.org/ghemical/
Gnumed	http://www.gnumed.org
Medical Dataserver	http://www.mii.ucla.edu/dataserver
Medical Image Analysis	http://sourceforge.net/projects/mia
Moby	http://biomoby.open-bio.org/
Olduvai	http://sourceforge.net/projects/olduvai/
Openclinica	http://www.openclinica.org
Openemed	http://openemed.org/
Openemr	http://www.oemr.org/
Oscarmcmaster	http://sourceforge.net/projects/oscarmcmaster/
Probemaker	http://probemaker.sourceforge.net/
Res Medicinae	http://resmedicinae.sourceforge.net/
Rtools	http://www.bioconductor.org
S2s	http://bioinformatics.org/S2S/
Sashimi	http://sourceforge.net/projects/sashimi/
Sbeams	http://www.sbeams.org
Alkahest	http://www.alkahest.org

Array-A-Lizer	http://bioinformatics.org/cgi-bin/cvsweb.cgi/arrayalizer/
Ball	http://bioinformatics.org/project/?group_id=506
Biococoa Biolibrary For Obj-C	http://bioinformatics.org/project/?group_id=318
Biomedical Informatics Research Network	http://www.nbirn.net
Biosignal Tools	http://sourceforge.net/projects/biosig
Brain Imaging Simulation System	http://sourceforge.net/projects/brainimage
Cehr Net	http://sourceforge.net/projects/cehrnet/
Clearhealth Practice Management System	http://sourceforge.net/projects/clearhealth/
Common Open Source Medical Objects	http://sourceforge.net/projects/cosmos
Compbio Perl Module Suite	http://sourceforge.net/projects/compbio/
Delila-Genome Protein Binding Analysis	http://bioinformatics.org/project/?group_id=296
Deltastat 2D Gel Analysis	http://bioinformatics.org/project/?group_id=371
Dicom.Pm	http://dicomperl.sourceforge.net/
Dicomparser	http://sourceforge.net/projects/dicomparser/
Disease Gene Profiler	http://sourceforge.net/projects/dgp/
Eidors 3D	http://sourceforge.net/projects/eidors3d/
Extractor For Esi Quadrupole Tof Tandem Ms Data Enabled For High Throughput Batch Processing	http://sourceforge.net/projects/protms/
Febrl	http://sourceforge.net/projects/febrl
Free Practice Management	http://sourceforge.net/projects/freepm/
Free2bind	http://sourceforge.net/projects/free2bind/
Fs Kit Analysis Of Force Spectra	http://galaxie.cgb.ki.se/galaxieEST.html
Gata	http://sourceforge.net/projects/geckoe
Gecko	http://www.cebitec.uni-bielefeld.de/groups/brf/software/gendb_info/license.html
Gene Ontology Api For Java	http://combio.cs.brandeis.edu/GeneNotes/index.htm
Genetdb Regulatory Networks Platform	http://bioinformatics.org/project/?group_id=207
Genquire Genome Browsing And Annotation	http://bioinformatics.org/project/?group_id=99
Gentle	http://gentle.magnusmanske.de/
G-Language Project	http://www.g-language.org/main.html
Hdbstat	http://www.soph.uab.edu/ssg_content.asp?id=1289
Hospital Scenario Generator	http://sourceforge.net/projects/hsg/
Imlib3d	http://imlib3d.sourceforge.net/
Internet Pathology Suite	http://sourceforge.net/projects/ipath/
Isys	http://sourceforge.net/projects/isys2/
Jaligner	http://jaligner.sourceforge.net/

Jatlasview	http://www.biomedcentral.com/1471-2105/6/47/abstract
Jklinika	http://sourceforge.net/projects/jclinic/
Knowtator	http://bionlp.sourceforge.net/Knowtator/index.shtml
Librarian Virtual Library Of Pdfs	http://bioinformatics.org/project/?group_id=131
Mccore	http://sourceforge.net/projects/mccore
Medical Words	http://sourceforge.net/projects/medicalwords/
Medslt	http://sourceforge.net/projects/medslt
Msatfinder	http://www.bioinf.ceh.ac.uk/msatfinder/
Mugen	http://genome.jouy.inra.fr/MuGeN/
Netepi	http://sourceforge.net/projects/netepi
Ondex Suite	http://ondex.sourceforge.net/
Open Infrastructure For Outcomes	http://www.txoutcome.org/
Openvista	http://www.pacifichui.org/OpenVista/
Osirix	http://sourceforge.net/projects/osirix/
Paclims	https://sourceforge.net/projects/paclims/
Paracelsus	http://sourceforge.net/projects/paracelsus
Patient Record Anonymization Program	http://sourceforge.net/project
Pentaplot	http://sourceforge.net/projects/pentaplot
Phaccs	http://sourceforge.net/projects/phaccs/
Ping	http://sourceforge.net/projects/pinghealth/
Portablecbl	http://sourceforge.net/projects/cbl
Prehospital Medical Information System	http://sourceforge.net/projects/premis/
Probepicker	http://sourceforge.net/projects/probepicker/
Protms	http://www.protein-ms.de
Pydaylight	http://www.dalkescientific.com/PyDaylight/
Real Time Medical Consultation System	http://sourceforge.net/projects/realmedic
Semantic Moby	http://biomoby.open-bio.org/index.php/semantic-moby/
Sequence Manipulation Suite 1	http://bioinformatics.org/project/?group_id=339
Sight	http://jsight.sourceforge.net/index_SF.htm
Simpacs	http://www.medical.toshiba.com/clinical/radiology/simpacs.htm
Smith-Waterman On Greentea	http://bioinformatics.org/project/?group_id=134
Sqlcare	http://sourceforge.net/projects/sqlcare/
Tassel	http://sourceforge.net/projects/tassel/
The Pogo Group	http://www.bioinformatics.org/pogo/
Tls Motion Determination	http://pymmlib.sourceforge.net/
W-Curve Genomic String Visualization	http://www.bioinformatics.org/wcurve/
Xml4mat Xml/Matlab Format Converter	http://bioinformatics.org/project/?group_id=172

3dspi	http://prisma.insa-lyon.fr/~kfrugier/index.php?page=3dspi
A Taxonomic Search Engine: Federating Taxonomic Databases Using Web Services	http://www.biomedcentral.com/1471-2105/6/48
A Two-Way Interface Between Limited Systems Biology Markup Language And R	http://www.biomedcentral.com/1471-2105/5/190
Abc	http://mendel.stanford.edu/sidowlab/downloads/ABC_GERP/abcgerp.html
Amc Post Processing Package	http://idoimaging.com/cgi-bin/imaging/program.pl?ident=254
Amide	http://amide.sourceforge.net/
An Edit Script For Taxonomic Classifications	http://darwin.zoology.gla.ac.uk/~rpage/forest/
An Imaging System For Standardized Quantitative Analysis Of C. Elegans Behavior	http://www.pubmedcentral.nih.gov/articlerender.fcgi?tool=pubmed&pubmedid=15331023
Anovarray	http://www.biomedcentral.com/1471-2105/6/150
Arb	http://www.arb-home.de/
Arraycghbase	http://medgen.ugent.be/arrayCGHbase/
Arrayd	http://www.igib.res.in/scientists/arrayd/arrayd.html
Assessing Local Structural Perturbations In Proteins	http://www.biomedcentral.com/1471-2105/6/226
Autofact	http://megasun.bch.umontreal.ca/Software/AutoFACT.htm
Autoprime	http://www.autoprime.de/
Biofloweb	http://urgi.infobiogen.fr/BioFloWeb
Biomap	http://proteinontology.info/
Bionets	http://x.amath.unc.edu:16080/BioNetS
Biotext	http://biotext.berkeley.edu/
Blast Score Ration Analysis	http://www.microbialgenomics.org/BSR/
Blox	http://gecco.org.chemie.uni-frankfurt.de/h-blox/hblox.html
Caadapter	http://ncicb.nci.nih.gov/download/
Caryoscope	http://caryoscope.stanford.edu/
Cghpro	http://www.molgen.mpg.de/~abt_rop/molecular_cytogenetics/ArrayCGH/CGHPRO/
Cgmim	http://www.biomedcentral.com/1471-2105/6/78/abstract
Codaln	http://www.bioinf.uni-leipzig.de/Software/codaln/
Dishevelled	http://www.pubmedcentral.nih.gov/articlerender.fcgi?artid=533865
Efficient Decoding Algorithms For Generalized Hidden Markov Model	http://www.pubmedcentral.nih.gov/articlerender.fcgi?tool=pubmed&pubmedid=15667658#N0x865898

Gene Finders	0.0x86eb7b8
Emboss-Gui	http://bioinfo.pbi.nrc.ca/~lukem/EMBOSS/
Emerencia	http://emerencia.math.chalmers.se/
Estima	http://titan.biotec.uiuc.edu/ESTIMA/
Expander	http://www.cs.tau.ac.il/~rshamir/expander/
Fact	http://www.factweb.de/
Fieldtrip	http://www2.ru.nl/fcdonders/fieldtrip/download.html
Forest	http://darwin.zoology.gla.ac.uk/~rpage/forest/
Galaxieest	http://www.pdbj.org/GASH/
Gash	http://bioinformatics.rcsi.ie/~redwards/gasp/
Gasp	http://gata.sourceforge.net/
Gendb	http://bioinformatics.org/project/?group_id=474
Genenotes	http://combio.cs.brandeis.edu/GeneNotes/index.htm
Geneorder Coregenes	http://binf.gmu.edu/genometools.html
Generalizations Of Markov Model To Characterize Biological Sequences	http://www.biomedcentral.com/1471-2105/6/219
Generating Quantitative Models Describing The Sequence Specificity Of Biological Processes With The Stabilized Matrix Method	http://www.mhc-pathway.net/smm
Genexplorer	http://www.biomedcentral.com/1471-2105/5/141
Genezilla	http://bioinformatics.org/project/?group_id=470
Genomeviz	http://www.biomedcentral.com/1471-2105/5/198
Genome-Wide Prediction Display And Refinement Of Binding Sites With Information Theory-Based Models	http://www.biomedcentral.com/1471-2105/4/38
Genomic Multiple Sequence Alignments: Refinement Using A Genetic Algorithm	http://www.biomedcentral.com/1471-2105/6/200
Go-Perl Amigo	http://search.cpan.org/~cmungall/go-perl/
Graphical Oncology Diagnostic System	http://www.cs.umb.edu/gods/
Gridstuffer	http://cmgm.stanford.edu/~cparnot/xgrid-stanford/index.html
Grock	http://egee-na4.ct.infn.it/biomed/GROCK.html
High Througput Snp Detection Pipeline	http://research.imb.uq.edu.au/seqdoc/
High-Throughput Gominer	http://discover.nci.nih.gov/gominer/htgm.jsp
Html4blast	http://bioperl.org/wiki/Module:Bio::Tools::Run::PiseApplication::html4blast
Htsnper1.0	http://bmc.ub.uni-potsdam.de/1471-2105-6-38/
Identitag	http://pbil.univ-lyon1.fr/software/identitag/

Integrated Web Service For Improving Alignment Quality Based On Segments Comparison	http://www.biomedcentral.com/1471-2105/5/98
Ipath	http://ipath.ch/team/brauchli
Kclms	http://puggy.symonds.net/~hareesh/kidwai/
Ldmas	http://molpath.his.path.cam.ac.uk/bioinformatics/LDMAS.shtml
Libcov	http://users.cs.dal.ca/~cblouin/libcov/
Mars	http://www.biomedcentral.com/1471-2105/6/101
Maxd	http://bioinf.man.ac.uk/microarray/maxd/
Mbetoolbox	http://www.biomedcentral.com/1471-2105/6/64#IDA0LZDJ
Mcgh	http://folk.uio.no/junbaiw/mcgh/
Mevislab	http://www.mevislab.de/
Modbiosql	http://mbiosql.biomembrane.hu/mbs_docs.htm
Moltalk	http://www.moltalk.org/
Mouseatlas Software	http://www.loni.ucla.edu/MAP/
Mprime	http://kbrin.a-bldg.louisville.edu/Tools/MPrime/
Multiclass Discoverer	http://www.thep.lu.se/~markus/software/classdiscoverer/
Muscle	http://www.drive5.com/muscle
Opensputnik	http://www.opensputnik.org
Paircomp Familyrelationsii And Cartwheel	http://family.caltech.edu/
Partigene	http://www.nematodes.org/PartiGene/
Pegasys	http://bioinformatics.ubc.ca/pegasys/
Permol	http://www.biologie.uni-regensburg.de/Biophysik/Kalbitzer/permol/permol.html
Phenotype And Disease Ontologies	http://diseaseontology.sourceforge.net
Phylogenetic Reconstruction Of Ancestral Character States For Gene Expression And Mrna Splicing Data	http://rossnes.org/phyrex/
Predicting Functional Sites With An Automated Algorithm Suitable For Heterogeneous Datasets	http://www.pmap.csupomona.edu/MINER/dl.html
Primermatch	http://bioinformatics.org/project/?group_id=415
Progenexpress	http://progenexpress.sourceforge.net/
Promi	http://promi.mpimp-golm.mpg.de/home.shtml
Prot4est	http://bioinformatics.org/cgi-bin/cvsweb.cgi/PartiGene/prot4EST/
Psi-Blast-Iss	http://www.ibt.lt/bioinformatics/iss/
Pyevolve	http://cbis.anu.edu.au/software.html#PyEvolve
Scompy	part of biopython
Seqdoc	http://research.imb.uq.edu.au/seqdoc/

Seqx	http://janbiro.com/Downloads.html
Simprot	http://www.uhnres.utoronto.ca/labs/tillier/software.htm#3
Spcr	http://moleco.sjtu.edu.cn/SPCR
Squid – A Simple Bioinformatics Grid	http://www.biomedcentral.com/1471-2105/6/197#B13
Ss-Wrapper	ftp://ftp.genome.uab.edu
Stam	The R Project for Statistical Computing
Stanford Microarray Database	http://genome-www5.stanford.edu/
Sting Millennium Suite	http://mirrors.rcsb.org/SMS/index_m_mirror.html
Storing Linking And Mining Microarray Databases Using Srs	http://www.erasmusmc.nl/gatcplatform
Superficial	http://bioinformatics.charite.de/superficial/
T.I.M.S	http://bioinformatics.org/macroshack/prog_list.html
Taverna	http://www.mygrid.org.uk/index.php?module=pagemaster&PAGE_user_op=view_page&PAGE_id=44&MMN_position=53:51:52
Tetra	http://www.megx.net/tetra
Text2knowledge Text Mining Tools	http://bioinformatics.org/textknowledge/
The Cartwheel Bioinformatics Project	http://cartwheel.caltech.edu/
The Indeval	http://www.sci.muni.cz/botany/elzdroje/indeval/
Tmb-Hunt	http://www.biomedcentral.com/1471-2105/6/56
Tools Enabling The Elucidation Of Molecular Pathways Active In Human Disease: Application To Hepatitis C Virus Infection	http://labs.systemsbiology.net/galitski/hepc/if_and_ct.html
Tools For Loading Medline Into A Local Relational Database	http://biotext.berkeley.edu/software
Toophpix	http://truth.positive-internet.com/~mpreston/tp/tp0412intro.html
Transalign	http://www.tierzucht.tum.de/Bininda-Emonds/
Trans-Proteomic Pipeline	http://tools.proteomecenter.org/TPP.php
Vestige	http://cbis.anu.edu.au/software.html
Vienna Rna Package	http://www.biomedcentral.com/1471-2105/6/89#IDAF2KLM
Viral Bioinformatics Resource Center	http://athena.bioc.uvic.ca/
Visualization And Analysis Of Microarray And Gene Ontology Data With Treemaps	http://www.cs.umd.edu/hcil/treemap/
Visualization Of Comparative Genomic Analyses By Blast Score Ratio	http://www.microbialgenomics.org/BSR/
Visualization-Based Discovery And	http://function.princeton.edu/ChARMView/charm_
