# Supplementary material for: A Survey of Quality Assurance Practices in Biomedical Open Source Software Projects
Source: J Med Internet Res. 2007 May 7;9(2):e8. doi: 10.2196/jmir.9.2.e8 (PMC1874720; doi:10.2196/jmir.9.2.e8)
Supplement: Supplementary file 3 [file jmir_v9i2e8_app3.pdf]

Multimedia Appendix 3. CHERRIES Summary

**Design**	Survey Design	Target population was the developers of OS biomedical projects. The projects were identified from the SourceForge, FreshMeat, and BioMedCentral Websites as well as the authors' own knowledge and that of colleagues. The names and contact information of the developers in these projects were obtained from the project websites, CVS, mailing list, and bug repositories.
**IRB approval and informed consent process**	IRB Approval	The study was reviewed and approved by the IRB of the University of Maryland, Baltimore County (UMBC).
	Informed Consent	At the beginning of the survey form, the participants were informed that their responses would be used only for the purpose of this research and their identity or contact information would not be shared. They were told that filling out this survey would take approximately 20-25 minutes.
	Data Protection	The online survey tool was installed on a server computer located at the researchers' lab at UMBC. This tool provides password-controlled access to its administrators, the researchers, to enter and view respondents' names and e-mail addresses.
**Development and pre-testing**	Development and testing	The survey was developed and tested using the OS tool, PhpSurveyor. The researchers tested this tool several times and assured its functionality and usability before the actual survey took place.
**Recruitment process and the description of the sample having access to the questionnaire**	Open vs. Closed	The survey was a closed survey. The survey tool automatically created e-tokens (long and complex URLs) that allowed access to the on-line survey form, and e-mailed them to the respondents. Each potential respondent received one token.
	Contact Mode	Respondents received e-mails explaining the goals and purposes of the survey and asking their contribution. E-mails included the tokens which took the potential respondents to the on-line survey form. After the first e-mail, reminder e-mails were sent over a period of six weeks for non-respondents after the first week.
	Advertising	No advertising was made.

**Survey Administration**	Web / E-mail	The contacts were made by e-mails. However, the survey was web-based. The respondents used their web-browsers to respond. The data was collected automatically after their submission on researchers' computer that hosts the web server and MySql database. All data kept in this database is password protected.
	Context	Following the special URL (token) given in the e-mail, the respondents were only able to view the survey form. They were not shown any other content.
	Mandatory/ Voluntary	The respondents were able to view the survey form without filling out the survey and submitting their answers. Responding to the survey was voluntary. Upon clicking on the submission button, it was checked whether the response was a complete response or not. The respondent was reminded and asked to answer in case any question was left unanswered.
	Incentives	No incentive was given other than telling respondents that they would be informed about any resulting report or publication of this research.
	Time/Date	The survey was conducted between Oct 10 and Nov 17, 2005.
	Randomization	No items or questionnaires were randomized.
	Adaptive Questioning	Adaptive or conditional questioning was not used.
	Number of Items	The survey questions relevant for this paper are shown in Appendix 1.
	Number of screens	The whole questionnaire was a single page, the respondents replied by scrolling down to the next question.
	Completeness check	Each submitted response was checked for completeness. This functionality was available in the survey instruments by making all of the questions mandatory.
	Review Step	The respondents could review their answers before submission by scrolling up the page.
**Response Rates**	Individual Response Rate	The individual response rate was 18.4% (the number of individuals responded / the number of individuals that had valid e-mail addresses and invited with personal tokens), and the project response rate was 46.3%
	Unique Site Visitor	Not available
	View Rates	Not known
	Participation Rate	Not known

	Completion Rate	100%
**Preventing multiple entries from the same individual**	Cookies used	Each respondent received a token which is a long and complex URL that can be used to complete the survey only once.
	IP check	Not used
	Log file analysis	Some e-mail addresses were not valid anymore. The e-mails sent to these addresses were returned, and they were detected from the e-mail logs of the root account of our server machine. We excluded these individuals in calculating our response rate.
	Registration	The user could view the survey page only until s/he submitted the completed survey. The survey was never shown again to this user with the token that he used.
**Analysis**	Handling of incomplete questionnaires	All of the survey forms were completed since the instrument checked for completeness and only accepted the complete forms.
	Questionnaires submitted with atypical time stamp	Time to fill out the survey was not tracked. However, respondents only had one opportunity to submit the survey with their e-token, after which that token was disabled.

9 References

1. Prada, G., et al., *Understanding Health Care Cost Drivers and Escalators*. 2004, The Conference Board of Canada.
2. Prada, G., et al., *Challenging Health Care System Sustainability: Understanding Health System Performance of Leading Countries*. 2004, The Conference Board of Canada.
3. Irving, R., *2003 Report on IT in Canadian Hospitals*. 2003, Canadian Healthcare Technology.
4. Raymond, B. and C. Dold, *Clinical information systems: Achieving the vision*. 2002, Kaiser Permanente Institute for Health Policy.
5. Bates, D., et al., *A proposal for electronic medical records in US primary care.* Journal of the American Medical Informatics Association, 2003. **10**(1): p. 1-10.
6. Goulde, M. and E. Brown, *Open source software: A primer for health care leaders*. 2006, California Healthcare Foundation.
7. Kantor, G., W. Wilson, and A. Midgley, *Open-source software and the primary care EMR.* Journal of the American Medical Informatics Association, 2003. **10**(6): p. 616.
8. Giera, J., *The Costs And Risks of Open Source*. 2004, Forrester.
9. Hunt, F., D. Probert, and S. Barratt. *Adopting new technology: The case of open source software at Marconi*. in *The 12th International Conference on Management of Technology (IAMOT 2003)*. 2003.
10. Dal Molin, J., *Open source software in Canada: A collaborative fact finding study*. 2003, e-cology Corporation.
11. Dedrick, J. and J. West. *An exploratory study into open source platform adoption*. in *Proceedings of the 37th Hawaii International Conferenec on System Sciences*. 2004.
12. Didio, L., *Linux, Unix and Windows TCO Comparison, Part 1*. 2004, The Yankee Group.
13. Valdes, I., et al., *Barriers to proliferation of electronic medical records.* Informatics in Primary Care, 2004. **12**: p. 3-9.
14. Kolata, G., *In unexpected Medicare benefit, US will offer doctors free electronic records system*, in *New York Times*. 2005.
15. Goldstein, D., S. Ponkshe, and R. Maduro, *Analysis of open source software (OSS) and EHRs: Profile of increasing use of OSS in the federal government and healthcare*. 2004, Medical Alliances Inc.
16. Elsner, C., et al., *Open source or commercial products for electronic data capture in clinical trials ? A scorecard comparison.* Computers in Cardiology, 2003. **30**: p. 371-373.
17. Erickson, B., S. Langer, and P. Nagy, *The role of open-source sofwtare in innovation and standardizatiuon in radiology.* Journal of the American College of Radiology, 2005. **2**(11): p. 927-931.
18. Gentleman, R.C., et al., *Bioconductor: open software development for computational biology and bioinformatics.* Genome Biology, 2004. **5**(10).
19. Stajich, J., *Bioperl project report*. 2002.
20. Pocock, M., *BioJava Toolkit Progress*. 2002.
21. McCormick, J. and D. Gage. *Cincinnati Children's Hospital: Shots in the Dark*. Baseline: The Project Management Center 2004 [cited 2006 1st December]; Available from: http://www.baselinemag.com/article2/0,1540,1655082,00.asp.
